# Undernutrition and Overnutrition Burden for Diseases in Developing Countries: The Role of Oxidative Stress Biomarkers to Assess Disease Risk and Interventional Strategies

**DOI:** 10.3390/antiox6020041

**Published:** 2017-06-08

**Authors:** Francesca Mastorci, Cristina Vassalle, Kyriazoula Chatzianagnostou, Claudio Marabotti, Khawer Siddiqui, Ahmed Ould Eba, Soueid Ahmed Sidi Mhamed, Arun Bandopadhyay, Marco Stefano Nazzaro, Mirko Passera, Alessandro Pingitore

**Affiliations:** 1 Clinical Physiology Institute, National Research Council, 56124 Pisa, Italy; mastorcif@ifc.cnr.it (F.M.); anubi@ifc.cnr.it (M.P.); pingi@ifc.cnr.it (A.P.); 2 Fondazione Regione Toscana G. Monasterio, 56124 Pisa, Italy; kyriazoula.chatzianagnostou@ftgm.it; 3 Cardiovascular Department, Ospedale della Bassa val di Cecina, 57123 Cecina, Italy; c.marabotti@gmail.com; 4 Department of Cardiology, Ruby General Hospital, Kolkata 700107, India; drsabinsid@yahoo.com (K.S.); alessandro.pingitore@ifc.cnr.it (A.B.); 5 Centre National de Cardiologie, Nouakchott 000, Mauritania; docteureba@live.fr (A.O.E.); ouldethmanesidimhamed@yahoo.fr (S.A.S.M.); 6 Cardiovascular Department, San Camillo Hospital, 00152 Roma, Italy; msnazzaro@gmail.com

**Keywords:** nutrition, epidemiological transition, non-communicable diseases, cardiovascular disease, inflammatory biomarkers, antioxidants, oxidative stress biomarkers

## Abstract

The increased life expectancy, urbanization, and unhealthy lifestyle characterized by a shift towards a sedentary lifestyle and decreased energy expenditure are considered the main drivers of epidemiological transition. In particular, developing countries are facing a double burden caused by coexisting under- and over-nutrition, which causes a change in the disease profile from infectious diseases to a chronic degenerative pattern. This review discusses the under- and over-nutrition context in Mauritania and India, two countries that are experiencing a nutritional transition, and where we began a collaboration with local medical staff to integrate interventional and diagnostic guidelines. If many studies about diet and its relationship to non-communicable diseases are available for India, very few nutrition and cardiovascular risk studies have been conducted in Mauritania. Presently, with the exponential increase of nutrition-related diseases, targeted approaches are needed to provide balanced diets in parallel with the development of national preventive health systems and screening programs adapted to local needs. In this context, the measurement of oxidative stress biomarkers could be promising as an additive tool to assess cardiovascular (CV) risk in general population, and ameliorating prevention in patients at CV risk or with overt CV disease. Moreover, the possibility of improving the outcome by the direct employment of antioxidant remains plausible. Moreover, studies on the content of antioxidant in different foods may be helpful to develop a balanced diet, and achieve the maximal nutritional and functional properties of cultivars with benefits for human health.

## 1. Introduction

Lifestyle habits, in general, surely including diet and exercise, can confer a risk to developing diseases across the human lifespan [1]. Nutrient supply and diet composition can exert immediate and/or long-term beneficial or adverse implications on health, and even nutrition deficiency in the pre-birth phase may affect late-life health outcomes, increasing the risk of non-communicable diseases (NCDs) [2].

Although under-nutrition issues associated to industrialization and urbanization have been overcome in developed countries, at least 75% of all deaths are attributable to unbalanced diet and over-nutrition, which cause an onset of NCDs. Nonetheless, this percentage is also increasing in developing countries (40% of the total deaths), due to the adoption of a Western diet and lifestyle. Thus, developing countries have to face a double burden of under- and over-nutrition, which need to be understood, in order to adopt preventive strategies and help developing societies to deal with this burgeoning problem [1].

According to the World Health Organization (WHO), NCD-related deaths are projected to increase by 15% globally until 2030, with most increases taking place in Africa, South-East Asia, and Eastern Mediterranean regions where they are expected to increase by more than 20% [3]. As developing countries have become wealthier, the population moves from a traditional diet high in carbohydrates and fiber and low in fat and sugar, to a typical Westernized diet, characterized by a higher intake in energy, saturated fat, sodium, sugar, and low in fiber, and associated with physical inactivity and other unhealthy lifestyle behaviors (e.g., smoking), which increase the risk of obesity, hypertension, stroke, type 2 diabetes, and ischemic heart disease (IHD) [4].

In addition, this change is pivotal for the epidemiological transition from a condition of predominance of nutritional deficiencies and infectious diseases, to those classified as degenerative chronic diseases (such as cardiovascular disease, cancer, and diabetes) [5]. Presently, different countries in the world, or even different regions within a country, are at different stages of the epidemiologic transition, exacerbated by a poor lifestyle, which includes decreased physical activity, a stressful lifestyle, high alcohol consumption, and tobacco use [6].

India and Mauritania are two countries in nutritional and epidemiological transition, in which the two faces of dis-nutrition coexist. Nonetheless, behind this common status, there are significant differences between these two countries. In fact, many studies are available in different Indian social classes, urban or rural areas, children or adults, which may represent a platform to plan targeted programs. Instead, there is a lack of scientific studies for Mauritania. This knowledge is essential to understand different social, religious, economic, and geographical characteristics. Thus, these two countries may be considered two examples of how nutritional deficiencies vary from country to country, and even from area to area in the same country, depending on socio-economic status. In light of this, efficient preventive strategies are needed and urgent measures should be taken to control risk factors, like tobacco, alcohol, obesity, blood pressure, diet, and inactivity. Nonetheless, it is necessary to collect enough data on specific contexts in order to adequately adapt international interventional programs to local needs.

This review reports the current nutritional and epidemiological evidences in India and Mauritania, where we began a close collaboration with local medical staff to integrate international interventional and diagnostic guidelines to local needs. Analogous approaches can also be adapted to other countries according to different local social, religious, economic, and geographical characteristics.

## 2. Oxidative Stress Status: Measures and Possible Intervention

Oxidative stress and free radical production are involved in the etiology of various chronic conditions, including cancer, atherosclerosis, neurological, and cardiovascular diseases [7]. As oxidative injury may act on different cellular components and substrates, lipids, proteins, and nucleic acids can be measured to estimate oxidative stress status [8]. However, conjugated dienes, hydroperoxides, malondialdehyde (MDA), 4-hydroxynonenal, hydrocarbons, such as pentane and ethane (in breath), F2-isoprostanes, and oxidized-low density lipoproteins, remain the most used biomarkers [8].

For the antioxidant counterpart, the total antioxidant capacity (TAC), rather than a single antioxidant, including enzymatic (e.g., catalase, glutathione peroxidase, superoxide dismutase), as well as non-enzymatic biomarkers (e.g., vitamins E, A, C, and glutathione and uric acid) can be evaluated [8].

Lifestyle habits (e.g., smoking, physical activity), as well as diet or nutritional supplementation, can modulate oxidative stress status, with possible effects on health [9,10,11]. In this context, there are some data available on antioxidant supplementation in India. A study conducted on Indian children aged 10–12 showed that 100 g/day of cauliflower leaf powder (taken for about four months) may improve hemoglobin, serum retinol levels, height, weight, and nutritional status [12].

In Indian adult, the antioxidant supplementation, administered as syrup (A-Z 5 mL b.i.d., predominantly composed of antioxidants, vitamins, and trace elements) was able to reduce MDA levels and increase zinc and erythrocyte superoxide dismutase capacity, although with no significant effect on vitamin E levels [13].

More recently, the efficacy of neonatal oral supplementation with vitamin A in reducing mortality (oral capsules containing vitamin A (retinol palmitate 50,000 IU plus vitamin E 9·5-12·6 IU) or placebo (vitamin E 9·5-12·6 IU) within 72 h of birth), was studied in two districts of Haryana, India. Data obtained indicated a modest reduction of mortality in the vitamin A-treated infants compared with the placebo group (656 versus 726) [14]. In these settings, regular antioxidant supplementation may help to improve the nutritional as well as antioxidant status by neutralizing free radical formation, followed by health protection. However, data are sparse and more additional evidences are required. Moreover, many unsolved issues on antioxidant exogenous supplemental remain, because effectiveness of antioxidants may vary according to many variables, as dose, duration of treatment, interaction with diet nutrients and drugs, the use of single or combined antioxidant molecules. In this context, an equilibrate diet with the consumption of various fruits and vegetables, whole grains, seeds, legumes and beans, as natural sources of antioxidants, is safer than supplementation, and it would be the final target of national health programs [15]. Measurement of some nutrition-related and oxidative stress biomarkers may be useful to assess the risk of diseases, monitor the effect of lifestyle changes, and act as potential interventional target to improve oxidative stress status.

## 3. The Islamic Republic of Mauritania

**Figure 1:**
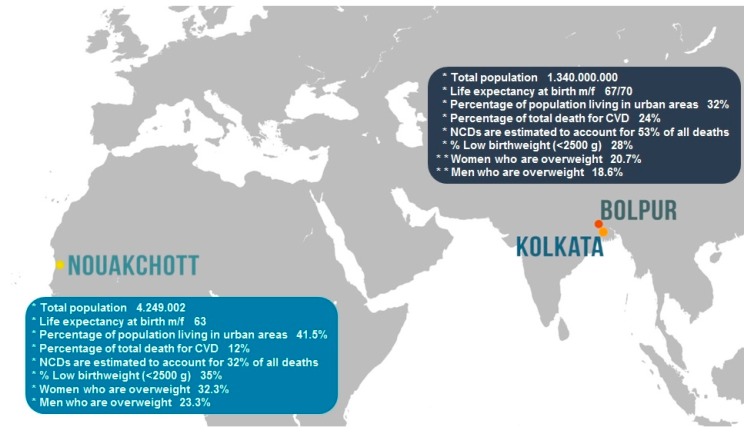
Country profiles by World Health Organization 2015–2016 Reports (*) and National Family Health Survey 2015–2016 India Fact Sheet (**). (CVD: cardiovascular disease; NCDs: non-communicable diseases).

Africa is a continent of great diversity, extending from highly-industrialized cities where people follow an urban Westernized lifestyle, to remote rural regions with traditional lifestyles; consequently, population reflects different stages of the epidemiological health transition according to geographical areas and rural or urban life. In Mauritania, life expectancy is around 60 years for both sexes (Figure 1). Twenty years of drought have caused a profound demographic transformation in this country, which is 90% desert without significant clusters of population [16]. The majority of the population lives in or around the coastal capital of Nouakchott and the city of Nouadhibou, and along the Senegal River in the southern part of the country [16]. The low-income economy depends on natural resources (e.g., fishing, iron, gold, copper, gypsum) and agriculture [16]. The epidemiological profile of Mauritania is still characterized by infectious diseases (e.g., malaria, tubercolosis). In addition, the percentage of children with congenital heart disease is high, as well as acquired heart disease among children with rheumatic fever, which is endemic in this country [17].

Moreover, nutritional issues related to under-nutrition still represent a great challenge for the lack of existing data. In particular, National programs have been promoted to collect, analyze, and improve data on the nutrition and health status in infants and children under two years of age in a group of six target African countries, including Burkina Faso, Chad, Mali, Mauritania, Niger, and Senegal [18]. Results from these studies evidenced that in these areas, the acute malnutrition prevalence remains high, diffusion of desirable nutrition related practices is low, and human resources available to carry out all nutrition-related program activities are largely insufficient [18].

The Mauritanian people faces serious health risks, many children suffer from diarrhoea, and other diseases related to deteriorating environmental conditions. About 2150 Mauritanians, including 1700 children under the age of 5 years, die every year from diarrhoeal disease [19]. In addition, according to the 2014–2016 Mauritania Humanitarian Needs Overview, 531,000 people (315,200 children) will require assistance, including 141,000 malnourished and 190,000 severely food-insecure, making necessary life-saving interventions, such as treatment for acute malnutrition. Only 464 out of 714 health facilities are currently providing integrated management services for acute malnutrition. However, it is also important to note that the epidemiological transition going on in Mauritania inevitably promotes diseases favored by physical inactivity, obesity, smoking, stress, hypertension, mostly prevalent in industrialized countries. A previous WHO survey, focusing on non-communicable disease in the Mauritanian general population, showed a 22.4% mean incidence of hypertension in both male and female, whereas the incidence of diabetes was around 6% [20]. Importantly, smoking was significantly higher in males, whereas obesity was present in females. These findings were confirmed by our study on patients with documented coronary artery disease (CAD) undergoing coronary revascularization (unpublished data). These data were obtained as part of the collaboration between Mauritania and Italy, started in 2011, when an Italian team of hemodynamic cardiologists (Italian Government program project AID 9580/ICU/MRT) went, for the first time, to the Centre National de Cardiologie of the capital Nouakchott. A cooperative effort was made with local physicians in order to teach coronary revascularization procedures as the main therapeutic option for treating the growing number of patients with acute myocardial infarction and symptomatic coronary artery disease (CAD). So far, 180 patients have been enrolled and underwent coronary angiography and percutaneous coronary revascularization for the following clinical conditions: recent acute or previous acute myocardial infarction or coronary syndrome (<6 months from the occurrence of the acute event), documented ischemia on effort and left ventricular dysfunction. Various studies identified oxidative stress among main contributors for the onset and development of the atherosclerosis process and CAD [11,21,22,23]. Moreover, high levels of oxidative stress have been associated with different CV risk factors, including cigarette smoking, obesity, diabetes mellitus, hypertension, physical inactivity, and hypercholesterolemia, contributing to endothelial dysfunction, and to the risk of CV mortality and adverse events in CAD patients [9,11,24,25,26]. Therefore, the evaluation of oxidative stress biomarkers could be employed as an additive tool to assess CV risk in primary, as well as secondary, prevention of Mauritanian patients.

Oxidative stress is not included in the current algorithm of the cardiometabolic risk, although it could improve the diagnostic and prognostic predictivity and help in the design of a strategy for prevention and management of CV diseases [27]. In particular, the addition of oxidative stress indices in a multimarker approach might allow the study of CV disease through many different mechanisms. Nonetheless, the optimal combination of biomarkers has not been defined yet and further knowledge in this research area is needed.

Moreover, the possibility to improve patient outcome by antioxidant interventions directed to reduce oxidative stress remain plausible, although currently inconclusive [23]. In fact, so far, antioxidant trials have failed to reduce the risk of CV disease. However, due to the complexity and diversity of mechanisms associated with oxidative stress, we believe that it is unlikely that non-targeted antioxidant therapy will easily prove to be effective. A possible alternative strategy might be to select patients under high oxidative stress, who would theoretically benefit the most from antioxidant treatment, thus representing ideal candidates to assess the efficacy of the antioxidant approach [23]. To this purpose, evaluation of baseline oxidative stress is essential. Other important determinants may be the dose and type (single or multiple) of antioxidant used and time of supplementation [7,23]. In addition, drugs interaction may be considered, because antioxidant treatment may blunt the effectiveness of drugs, such as statins and niacin [8]. All of these issues must be considered when planning future studies.

The knowledge of the relationship between socioeconomic and cardiovascular risk factors at the time of the epidemiological transition is a crucial step in developing scientific-based guidelines for national and global policies and priorities. Data from the WHO 2015 Reports indicate that one woman out of three is overweight/obese, and that 35% of babies are underweight; interestingly, both factors are related to the increase of oxidative stress and cardiovascular risk (Figure 1). In this regard, so far, no study reported oxidative stress and inflammatory biomarkers in the Mauritanian population, neither their correlation with diet, cardiovascular risk factors, and diseases. In our opinion, the complete lack of data in the field of nutritional deficiencies, as well in the adoption of incorrect dietary habits, must be evidenced to overcome the gap of knowledge in this field. In this setting, oxidative stress parameters can be also assessed as potential useful tools to estimate the efficacy of dietary intervention and of supplemental antioxidants. For example, in their study, Lemine et al. focused on the evaluation of six date palm cultivars commonly grown in Mauritania and largely consumed by the local population [28]. They evidenced that phenolics, as effective natural antioxidants, represented the major contributor of their natural antioxidant activity, thus being potentially beneficial to the health and wellbeing in Mauritanian people [28].

## 4. India

India is a country in rapid demographic transition. Life expectancy is increasing, and the share of the population above 60 years of age is growing at a rapid rate. Presently, people over 60 years of age are expected to live at least 75 years [29]. However, the population growth rate is not uniform in all states or regions of the country and government must face problems related to overpopulation, environmental pollution, diffuse poverty, and under-nutrition (Figure 1). India is within the nutritional transition phase. This is also suggested by preliminary and unpublished data of our study, performed in Kolkata children, living in slums, versus children belonging to the middle-high social class. Specifically, different anthropometric nutritional parameters, including mid-upper arm circumference (MUAC) and Z scores (weight for age, WAZ; height for age, HAZ; body mass index for age, BAZ), were significantly higher in children belonging to the middle-high social class, with respect to children living in the low social class.

**Figure 2:**
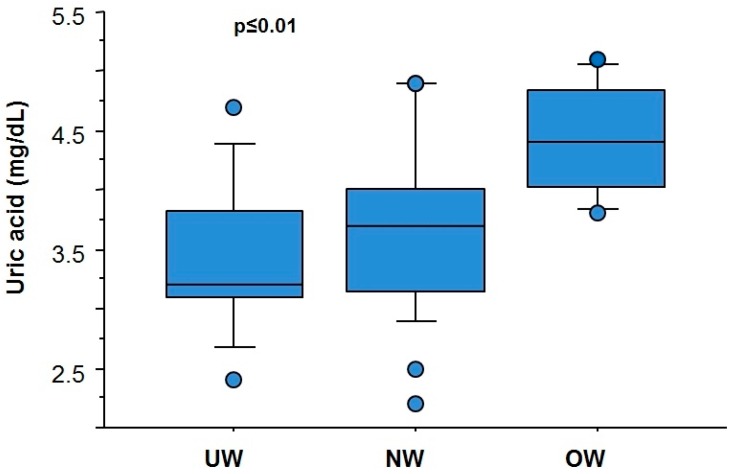
Levels of uric acid (UA) in under-, normal-, and over-weight (UW, NW, and OW, respectively) Indian children living in Kolkata.

In this context, we evaluated the levels of uric acid (UA), which is a potent antioxidant, but also identified as a cardiovascular risk factor if present at high levels [30]. In fact, experimental and in vitro findings evidenced that UA may act as an antioxidant to pro-oxidant, according to the level and microenvironment conditions [8]. Food intake may significantly affect the development of hyperuricemia. In fact, a low-fat dairy diet, vegetables, nuts, and legumes, lower UA, whereas a high intake of red meat and seafood, sugar sweetened drinks, processed foods and snacks increase the risk of hyperuricemia [30], mainly worsened by the content of fructose [30]. Thus, the spread of Western diet (rich in high fats and fructose content), street-food consuming (with high content in saturated fats and poor in fibers, vitamins and antioxidants) and sedentary habit may favor hyperuricemia. Accordingly, we found that UA was higher in Indian children that habitually consume street food (4.2 ± 0.6 vs 3.6 ± 0.7 mg/dL, *p* < 0.05), and progressively increase from under- to normal- and over-weight conditions in the overall population (3.4 ± 0.6, 3.6 ± 0.7, 4.4 ± 0.5 mg/dL, respectively: *p* ≤ 0.01; Figure 2).

By contrast, in low classes, under-nutrition is a consistent social plague both for adults and children, both in urban slums and rural areas [31,32,33,34]. In our study conducted in Bolpur (a rural area of West-Bengala) on children (aged 6–10 years), 36% of females and 67% of males resulted in being under-weight (15% and 43% severely under-weight, respectively; unpublished data), according to their WAZ and assessed growth using the WHO Growth Charts. A possible explanation could be maternal nutritional deprivation, leading to low-birth weight. This fact, associated with malnutrition during the first years of life, may drive to an increased risk of cardiometabolic disease and other NCDs in adulthood [35,36,37,38,39,40,41]. On the other hand, the diffusion of a typical Western diet, with decreasing intake of cereals, fruits and vegetables, an increasing intake of meat products and salt, coupled with declining levels of physical activity due to rapid urbanization, have resulted in escalating levels of obesity, dyslipidemia, subclinical inflammation (low grade chronic inflammation, characteristic of cardiometabolic disease), metabolic syndrome, type 2 diabetes mellitus, and coronary heart disease in Indians [42]. In this context, evidence of epidemiological transition in India show an increase in premature deaths in adults for NCDs, particularly in the urban areas [43].

For what concerns oxidative stress, Kolkata female rag pickers (median age: 30 years) had elevated serum level of inflammation, oxidative stress, platelet hyperactivity, and hypertension, with respect to housemaid controls [44]. Moreover, increased levels of serum MDA and significant depletions in the levels of vitamin E, zinc, and erythrocyte superoxide dismutase were found in Indian patients with severe acute malnutrition [13]. This situation was improved by supplementation of antioxidants for one month (syrup, A-Z 5 mL b.i.d.), which significantly reduced levels of MDA, while significantly increased zinc and erythrocyte superoxide dismutase capacity [13]. In addition, some life-style habits, such as regular cooking with biomass may increase particulate pollution in indoor air, inflammation, and oxidative stress biomarkers in blood, which correlate with higher blood pressure [45]. On the other hand, oxidative stress biomarkers are increased also by over-nutrition, as observed in prediabetes, diabetes, and obesity in different Indian adult cohorts [46,47,48]. The same findings are found in Indian children, where obesity induced high levels of MDA, C reactive protein, and UA, whereas malnutrition significantly decreased zinc, TAC, alkaline phosphatase, and albumin levels and increased MDA levels [49].

## 5. Conclusions

Under- and over-nutrition are the major common challenges in developing countries, with enormous impact on social, economic, and health care systems. Mauritania and India, although distant from the geographical point of view, transcend the stages of nutritional transition as symptoms of under-nutrition and over-nutrition coexist in the population. Thus, whether many diseases secondary to food deficiencies decrease due to improved nutritional conditions, a fast increase in the prevalence of other over-nutrition-related diseases are observed. In both countries, the prevalence of malnutrition remains high, the overall prevalence of desirable diet-related practices is low, and human resources are reportedly insufficient to carry out all nutrition-related programs.

For example, the percentage of children aged <5 years underweight correspond to 29% and 36% in urban and rural areas of India, respectively, and 20% in Mauritania (World Health Organization 2015 Reports and the National Family Health Survey 2015–2016 India Fact Sheet, [50,51]. With epidemiological transition, various factors considered in the calculation of CV risk adopted in Western countries must be investigated, taking also into account other potential specific territorial peculiarities, related to geographical, social, and religious aspects. In this context, the evaluation of some nutrition-related biomarkers, and the measurement of oxidative stress parameters may be useful to assess disease risk and act as potential interventional target for various disorders. Moreover, studies on the content of antioxidant in different foods may be helpful, in order to define a balanced diet, and achieve the maximal nutritional and functional properties of cultivars with benefits to human health.
